# History of canids in Chile and impacts on prey adaptations

**DOI:** 10.1002/ece3.7642

**Published:** 2021-07-08

**Authors:** Benjamín Silva Rochefort, Meredith Root‐Bernstein

**Affiliations:** ^1^ Facultad de Ciencias Biológicas Universidad Católica de Chile Santiago Chile; ^2^ Center of Applied Ecology and Sustainability Santiago Chile; ^3^ Institute of Ecology and Biodiversity Santiago Chile; ^4^ UMR CESCO CNRS Musée National d'Histoire Naturelle Paris France

**Keywords:** ambush, antipredator defense, cursorial, ethnography, evolution of prey behavior, hunting, predation strategy, prehispanic dogs

## Abstract

Artiodactyl prey species of Chile, especially guanacos (*Lama guanicoe*), are reported to be very susceptible to predation by pack‐hunting feral dogs. It has been previously suggested that guanacos and endemic South American deer may have evolved in the absence of pack‐hunting cursorial predators. However, the paleoecology of canid presence in southern South America and Chile is unclear. Here, we review the literature on South American and Chilean canids, their distributions, ecologies, and hunting behavior. We consider both wild and domestic canids, including *Canis familiaris* breeds. We establish two known antipredator defense behaviors of guanacos: predator inspection of ambush predators, for example, *Puma concolor*, and rushing at and kicking smaller cursorial predators, for example, *Lycalopex culpaeus*. We propose that since the late Pleistocene extinction of hypercarnivorous group‐hunting canids east of the Andes, there were no native species creating group‐hunting predation pressures on guanacos. Endemic deer of Chile may have never experienced group‐hunting selection pressure from native predators. Even hunting dogs (or other canids) used by indigenous groups in the far north and extreme south of Chile (and presumably the center as well) appear to have been used primarily within ambush hunting strategies. This may account for the susceptibility of guanacos and other prey species to feral dog attacks. We detail seven separate hypotheses that require further investigation in order to assess how best to respond to the threat posed by feral dogs to the conservation of native deer and camelids in Chile and other parts of South America.

## Graphical abstract

South American artiodactyls seem to be highly susceptible to feral dog predation, presenting a major conservation threat—why? We synthesize evidence as to whether feral dogs represent the first group‐hunting cursorial predator in regional evolutionary history. We present seven hypotheses that need to be investigated in order to fill evidentiary gaps, resolve evolutionary ambiguities, and understand how to make conservation interventions to protect South American camelids and deer.

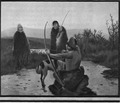

## 1 INTRODUCTION

The damage to biodiversity caused by feral and free‐ranging dogs *Canis familiaris* is well‐established globally. We use feral to mean dogs not socialized to humans, and free‐ranging to mean dogs fed by or scavenging off humans but not controlled or confined by humans. Young et al. (2011), in a global review of free‐ranging and feral dog impacts, show that the negative effects of *C. familiaris* on native species are widespread. Feral and free‐ranging dogs are a serious problem for conservation, mainly because of their predation on native species, transmission of diseases, and competition with other predators, which may destabilize ecosystems (Hughes & Macdonald, 2013; Ritchie et al., 2014). For example, on the Galapagos Islands a significant decline in marine iguanas (*Amblyrhinchus cristatus*) resulted from the establishment of dogs on the island (Kruuk & Snell, 1981). Feral and free‐ranging dogs in Brazilian protected areas displace native predators, spread diseases, and predate small‐ and medium‐sized wildlife (Lessa et al., 2016). South America is a hot spot of *C*. *familiaris* negative impacts on threatened species (Doherty et al., 2017). Why exactly are dogs such a destabilizing conservation threat in South America in particular? Geist (1998) partly addressed one angle of this question when he concluded that the South American deer are not adapted to cursorial predators such as dogs and that deer traits such as body size, locomotion, and reproductive investment, imply that there must have been only ambush predators present during the radiation of the South American deer species. In this paper, we re‐examine and extend this hypothesis, to ask whether it can explain why deer and also camelids of South America are so vulnerable to feral and free‐ranging dogs. While identification of any particular dog as feral or free‐ranging is often ambiguous, our focus is not on dog identity but on how they hunt, and in the contemporary conservation context, how they hunt without humans. We look more completely at all the evidence for the hunting strategies of prehistoric predators as well as domesticated canids associated with pre‐Columbian human presence. We focus on the case of Chile, and we end our review by evaluating the state of knowledge and suggesting hypotheses that need to be researched in order to better understand how the evolution and ecology of predator–prey interactions affect possible responses to the conservation challenge posed by feral and free‐ranging dogs.

In Chile, feral and free‐ranging dogs represent economic and environmental damage. *Canis familiaris* is estimated to cause 57,000 sheep deaths annually (Bonacic & Muñoz, 2014), although it should be noted that this includes deaths caused by both feral and nonferal dogs allowed to freely roam agricultural areas. Both feral and free‐ranging dogs cause significant reductions in the populations of threatened species in Chile. Free‐ranging dogs that are poorly fed are more likely to prey on and harass wildlife (Silva‐Rodríguez & Sieving, 2011). Rural dogs are interference competitors with the native fox *Lycalopex griseus* (Silva‐Rodríguez, Ortega‐Solís, et al., 2010; Silva‐Rodríguez, Verdugo, et al., 2010), eat the eggs of migratory birds that nest in Chile (Schüttler et al., 2009), and predate on native deer species, including pudu *Pudu puda* (Silva‐Rodríguez et al., 2010; Silva‐Rodríguez & Sieving, 2012) and huemul *Hippocamelus bisulcus* (Corti et al., 2010; Flueck & Smith‐Flueck, 2006). Reports from Peru (Barrio, 2007) state that fawns of taruka, *Hippocamelus antisensis*, a deer also found in northern Chile, may be killed by shepherd and feral dogs. The native camelids, principally guanaco (*Lama guanicoe*), are considered by experts (pers. comms. WCS Chile Grupo Núcleo Guanacos, 2020) to be particularly susceptible to being killed by feral or free‐ranging dogs, and dog presence is considered to be a factor preventing the conservation and reintroduction of *L. guanicoe* in large parts of its historical range (Farías et al., 2010; González, 2010). According to McLaren et al. (2018), vicuña (*Vicugna vicugna*), a high‐altitude relative of the guanaco, are hunted and harassed by humans with dogs, but dogs hunting in the absence of humans primarily predate on young offspring. It should be noted that the extent of the threat to species posed by dogs in Chile is partly due to the lack of a legal or cultural basis for their control, lethal or otherwise.

However, there are also behavioral and evolutionary features of predators to consider when trying to understand why feral and free‐ranging dogs present a large and difficult‐to‐control threat to Chilean wildlife. When understanding predation, there are two key factors to consider: the predator's niche and its hunting strategy. In terms of their niches, in general most canids (Carnivora: Canidae) are omnivores that do not kill (but may scavenge) prey larger than themselves (Sheldon, 1992). Small canids <6 kg are solitary foragers, canids 6–13 kg are facultative cooperative foragers, and larger ones are obligate cooperative foragers (Sheldon, 1992) likely to be specialized as carnivores, with some exceptions which we discuss in detail below (Wang et al., 2004). Canids, like other predators, may use either cursorial (pursuit) or ambush strategies. In cursorial strategies, the predator relies on its running adaptations and stamina to exhaust the prey and may lack specific killing tactics, rather relying on bringing the prey down by biting (Fox, 1987; González, 2010; Sheldon, 1992). In ambush strategies, the predator stalks the prey, remaining undetected until it can pounce on or rush at the prey from a short distance; it may rely on specialized killing tactics (Fox, 1987; Sheldon, 1992). For example, puma (*Puma concolor*) are ambush predators that kill with a specialized crushing bite to the head (Sarno et al., 1999).

In turn, prey species have adaptations such as scanning for predators, alarm calls, escape tactics, and group defense tactics, which may be suited for different predator body sizes, group sizes, and either pursuit or ambush predation strategies (see Caro et al., 2004). Essentially, to escape cursorial predators, prey need to outrun or lose them. By contrast, the advantage of ambush predators is their ability to take a targeted prey individual by surprise from a short distance. An effective defense against ambush predation is “predator inspection” which consists of staring at, approaching, surrounding, and/or following the predator so that it cannot gain this advantage (FitzGibbon, 1994 provides an excellent review). In a study of gazelle, FitzGibbon (1994) found that they were much more likely to inspect ambush than cursorial predators. Inspecting cursorial predators is likely to be risky and maladaptive, as by shortening the distance to the predator it may increase the chances of being overtaken.

In its feral or free‐ranging state, *C*. *familiaris* hunts in packs using a cursorial strategy (Farías et al., 2010; Ritchie et al., 2014). Geist (1998) notes that all of the South American deer lack appropriate defenses against what he calls “culling” predators, by which he appears to mean a cursorial strategy where one or more predators pick out and run down their prey. Geist notes that South American deer are “curious” which suggests they approach possible threats. They also respond too late to attacks, are saltatorial bounders rather than runners, and lack stamina (Geist, 1998). It has also been suggested that the guanaco lacks adaptations to escape cursorial pack hunters (Root‐Bernstein & Svenning, 2016). The guanaco demonstrates group vigilance and predator inspection, suitable for countering its main native predator, the puma (*P*. *concolor*), which hunts by solitary ambush (Darwin, 2015; Marino & Baldi, 2008; pers. obs. MR‐B). It is not entirely clear whether guanacos inspect dog packs, but González (2010) attributes high dog‐caused mortality to guanacos' short bursts of fast flight, leaving them unable to outrun dogs with greater stamina. Oyama (2006) describes vicuña (*V. vicugna*) in the Peruvian Andes as approaching possible threats such as dogs, and facing predators while giving an alarm call. Similarly to the case of guanaco, vicuñas' main predators, including puma and foxes, hide in the long grass and ambush them.

If South American deer and camelids lack appropriate defense behaviors, does this imply that this is an evolutionary problem—that they lack an adaptive trait? Although prey must respond differently and appropriately to different predators, this does not necessarily imply the evolution of completely independent responses to each predator. Lind and Cresswell (2005) make the valuable point that “antipredation behaviours are a composite of many behaviors that an animal can adjust…” (p. 945). Components of antipredator behavior can be lost under loss of predation pressure, but the presence of any kind of predator may be enough to retain a whole suite of antipredation behaviors for tens of thousands of years (Blumstein, 2006). So, to assess the hypothesis that key native Chilean prey species—camelids and deer—lack evolutionary adaptations to cursorial group predation, we would need to establish that they never in the past experienced selection pressures in the form of cursorial group‐hunting predators. Thus, we need to understand when cursorial group‐hunting canids first appeared in the southern cone of South America, which is not entirely clear. We focus on canids simply because there are not, and have never been, any group‐hunting or cursorial felids in South America.

It is clear that European conquistadors brought domestic dogs to the Americas starting around 500 years ago. However, the history of canids in South America, and ecological and behavioral descriptions of any kinds of prehispanic South American canids, has not been synthesized in a way accessible to conservationists. Here, we review existing evidence for the presence of prehispanic canids and their behaviors and ecological roles, focusing on Chile and the southern cone of South America.

## 2 CANIDS AND THEIR HUNTING ECOLOGY

### 2.1 South American canids

Up to 16 extinct canids (Wang et al., 2004), depending on the criteria used for phylogenetic studies, inhabited South America since around 3 Mya. Canids originated in North America and spread first to Eurasia upon the formation of the Bering Strait in the late Miocene (7–8 Mya), where they radiated into multiple lineages. Later, at 3.5 Mya they also spread to South America, in the Great American Biotic Interchange (GABI), which, however, may have started as early as 20 Mya (Bacon et al., 2015; Jaramillo et al., 2017; O'Dea et al., 2016). The GABI led to the evolution of new canid species, of which at least seven were hypercarnivores (Prevosti & Forasiepi, 2018). Hypercarnivores are those species with a diet composed of at least 70% meat (Holliday & Steppan, 2004), and so have unique ecological characteristics and requirements, distinct from other species of the group. Particularly in Canidae, hypercarnivores are large in size due to energetic constraints (Wang et al., 2004) and thus also very likely to be obligatory cooperative group hunters (Sheldon, 1992). The South American ancient hypercarnivores came from four genera, *Theriodictis*, *Protocyon*, *Speothos*, and *Canis*, the latter having three species, *C. gezi*, *C. nehringi*, and *C. dirus* (the dire wolf, also found in North America, although it has been moved to the genus *Aenocyon* as of recently—Perri et al., 2021) (Prevosti, 2010; Wang et al., 2004). Members of these genera all went extinct at the end of the Pleistocene, except the bush dog (*Speothos venaticus*) which is a small, short‐legged group‐hunting carnivorous canid living near water in the tropics. The hypercarnivorous Pleistocene species, distributed as far south as Argentina (Prevosti, & Forasiepi, 2018), would most likely have hunted megafauna. *Protocyon troglodytes*, a group‐living cursorial predator, and the non‐hypercarnivorous canids *Dusicyon* spp. in Uruguay hunted camelids, giant sloths, and other large‐ and medium‐sized prey (Prevosti et al., 2009). *Dusicyon* spp. went extinct much later than the hypercarnivores.

The 13 contemporary South American canids are divided into two lineages (Perini et al., 2010), one of species similar to Old World foxes (genera *Lycalopex*, *Atelocynus*, and *Cerdocyon*) and the other of species similar to Old World wolves (*Chrysocyon* and *Speothos*). Although it is unclear, some authors (Austin et al., 2013; Slater et al., 2009) consider this last lineage to be related to the extinct genus *Dusicyon* mentioned previously.

### 2.2 South American canids in Chile

The only one of the now‐extinct South American Pleistocene canids known to have had a distribution in Chile was *Dusicyon avus*, found in Chilean Patagonia and Tierra del Fuego, on the southern tip of Chile (e.g., Méndez et al., 2014; Prevosti et al., 2009). The genus *Dusicyon*, to which *D*. *avus* and *D. australis* (also known as the Falklands wolf) belonged, went extinct fairly recently, although the extinction process may have been initiated by reductions in its distribution beginning in the Pleistocene (Austin et al., 2013; Prevosti et al., 2011; Prevosti et al., 2015). In spite of this, there have been reported sightings of *D. avus* by English explorers as late as 1870. There are also theories that *Dusicyon* spp. underwent selection or hybridized with European *C*. *familiaris* and thus became unrecognizable but not necessarily extinct (Borrero, 2009). The English natural historian Charles Hamilton Smith wrote in 1839 that mainland *Dusicyon* spp. were tamed and used for hunting by South American tribes (quoted in Clutton‐Brock, 1977). *D. australis*, restricted to the Falklands Islands, and extinct by the 1880s, shows morphological traits typical of domesticated canids, such as a bulbous head and white patches on its pelage (Clutton‐Brock, 1977), all of which may be a consequence of a long interaction with humans. As humans arrived from in the Americas at the end of the Pleistocene, and in southern Chile around 14.5 kya (Braje et al., 2020; Gómez‐Carballa et al., 2018), this gives any *Dusicyon*–human relationship considerable potential historical depth.

The other South American Pleistocene canids, especially the hypercarnivorous species, are not known to have occurred in Chile (Carrasco, 2009). Since the uplift of the Andes occurred around 14 Mya (Le Roux, 2012), well before the GABI, it may have formed a barrier to their dispersal. At the same time, nondetection does not imply absence, and dire wolves are known to have descended down the west side of the Andes as far as Bolivia (Perri et al., 2021; Wang & Tedford, 2008). Still, the hyperarid regions between Bolivia and north‐central Chile may have formed a dispersal barrier to travel south into Chile. New evidence could change known distributions: Part of a skeleton of a large canid has been found in the Pampa de Tamarugal (Region of Tarapacá, northern‐most Chile), undated but conjectured to be from before the arrival of the conquistadors to Chile (C. Latorre pers. comm. 2018, N. Villavicencio pers. comm. 2020). Other Pleistocene carnivores, including *Smilodon populator*, *Panthera onca*, and *Arctotherium tarijense*, are, like *D*. *avus*, found only in the extreme south of Chilean Patagonia (Carrasco, 2009) where large parts of the Andean cordillera are below 1,000 m and thus less of a barrier to dispersal. Indirect evidence suggests that they also traveled further north into northern Patagonia at least (Labarca et al., 2014). Other large predators (*P*. *concolor*) were (and are still) found in central Chile (Nielsen et al., 2015).

Currently, three native canids exist in Chile, all of the genus *Lycalopex* (*L. culpaeus*, *L. griseus*, and *L. fulvipes*). These species are omnivores, consuming small prey (Iriarte, 2007). They have also been reported to opportunistically hunt juvenile guanacos (Novaro et al., 2009) in the Karukinka Natural Park, in Tierra del Fuego, where there are no pumas and the population of guanacos is high. According to Novaro et al. (2009), culpeo foxes *L. culpaeus* are cursorial predators. Juvenile guanacos, though much larger than culpeo foxes, are recorded to have been chased by them in two cases, and in at least one case, the attacking fox was kicked by several adult guanacos (Guzmán, 2009). *Lycalopex culpaeus* also prey on huemul (*Hippocamelus bisulcus*) fawns (Corti et al., 2010). It is thus likely that *Lycalopex* can exert a cursorial hunting selection pressure on juvenile large prey species, but since juveniles are often protected by adults who may attack the fox, this may mitigate the selection pressure. Juvenile antipredator responses might also be adaptations distinct from adult behaviors. Franklin et al. (1994) report that guanacos and llamas respond to coyotes, a slightly larger canid found in North America, by alerting to them, alarm calling, walking or running toward them, chasing them, kicking, and stomping on them.

### 2.3 South American dogs

Though *C. familiaris*, which coevolved along with humans in Eurasia, is found early in the archeological record of North America, probably as a result of Bering Strait crossings, they arrive much later in South America. Dogs are reported from 4.5 kya in Ecuador and the coast of Peru (Salomon & Stahl, 2008; Stahl, 1984, 2012), and up to 10 kya in other parts of the Americas (Allison et al., 1982; van Asch et al., 2013).

It seems most probable that any dog present in Chile arrived along migratory or trade routes from the Andes, with the earliest evidence of dogs in the form of artistic representations from cultures such as the Moche (1,900–1,300 ya) or the Chimú (1,100 kya–500 ya) (Vásquez Sánchez et al., 2009). Prates, Di Prado, et al. (2010) and Prates, Prevosti, et al. (2010) hypothesize that the presence of dogs in South America was mainly related to complex societies, such as the Andean societies of Peru and Ecuador previously mentioned, and that their arrival in the Southern Cone is related to an increase in long‐distance communication and trade by egalitarian hunter‐gatherer societies of the Pampas and Patagonia. The authors come to these conclusions based on archeological deposits in northern Argentina dating from around 1,000 ya (around 500 years before the arrival of the Spanish), which accords with other archeological evidence of early dog presence in southern Brazil (1,600 ya, Guedes Milheira et al., 2017) and in Uruguay (undated remains, but found in multiple‐use structures starting around 1,000 ya, López Mazz et al., 2018).

Many morphotypes of dogs may have been present in South America at different times. Gilmore (1950) lists nine possible breeds of South American dogs, remarking that parallel selection pressures or morphological constraints resulted in a terrier‐like dog (the Fuegian dog), a setter‐like dog (the Ona dog), a foxhound/greyhound type dog (the Tehuelche dog), and another terrier‐like dog (the Techichi dog), as well as hairless dogs. The Pre‐Columbian origins of these possible breeds are unknown. Van Asch et al. (2013) confirmed the pre‐Columbian origins of several formally recognized American breeds, including the Peruvian *perro sin pelo* (“hairless dog”). Vásquez Sánchez et al. (2009) summarize many possible morphotypes of South American dogs, including a short‐nosed dog from the Chicama Valley, the medium‐sized “helping dog,” the “pet” dog with a long body and short limbs, the miniature dog similar to a chihuahua, the hairless dog, a short‐haired dog, a Peruvian bulldog, a Peruvian sausage dog, a long‐haired Incan dog, and the Chiribaya shepherd used to herd llamas. Vásquez Sánchez et al. (2009) also discuss dogs depicted on Moche pots, including a morphotype consisting of a small‐ or medium‐sized spotted dog with a bulbous head, which appears in a deer‐hunting scene among other contexts. They speculate that this kind of dog might have been used to corner deer during hunting. Like the Peruvian pitbull (Cossios, 2018), many morphotypes may have been bred locally for certain periods of time, but later allowed to outcross with other morphotypes.

It is unclear whether prehispanic dogs of South America would have formed significant feral or free‐ranging populations, although large packs of feral or free‐ranging dogs are reported by the 18th century (Prevosti et al., 2015), and it is hypothesized that feral or free‐ranging populations existed soon after the beginning of dog domestication in Eurasia (Boitani & Ciucci, 1995). Dogs, when feral or free ranging, are omnivorous opportunists (Campos et al., 2007). Dogs that are fed or accidentally subsidized (e.g., through waste) by humans tend to predate much less on wild prey, although this depends on the nutritional quality of the food obtained from humans (Vanak & Gompper, 2009). An increase in feral or free‐ranging dog populations following European colonization may have increased dog predation pressure on guanacos and native deer.

### 2.4 South American dogs in Chile

Chile can be grouped into three large cultural areas: the North, with Incan influence, the Central‐South zone, dominated by the Mapuche peoples, and the Southern region, associated with coastal hunter‐gatherer cultures. We discuss South American dogs in Chile in the sequence of these three cultural areas from north to south. However, the evidence for prehispanic dogs in each zone is relatively weak.

In Arica (northern Chile), eight mummified dogs, dated to circa 1,450–500 ya, were found by Allison et al. (1982), belonging to the San Miguel, Inca, Cabuza, Alto Ramirez, and Maitas Chiribaya phases and periods. These are terrier‐like dogs 46–52 cm high, apparently similar in form to the Moche hunting dogs discussed by Vásquez Sánchez et al. (2009).

It is commonly held in Chile, as well as recorded by historians (Vial, 2010) and the ethnographer Ricardo Latcham (1822) that the contemporary Mapuche indigenous people of the center‐south of Chile previously had two words for one or more kinds of dogs that they had: *thegua* or *tregua* and *munutru*. Mapuche dogs may have been obtained from Argentinian Patagonian pre‐Columbian populations (Prates, Di Prado, et al., 2010; Prates, Prevosti, et al., 2010) or from the Inca (Uribe & Sánchez, 2016). According to Latcham (1822, p. 62), *munutru* refers to a dog with long curly hair on its face, or any “small ugly dog,” similar to what is today called a “quiltro,” usually translated as “mutt,” (ibid p. 62), and is reported by contemporary Mapuche people to mean anything with an “ugly face” (Loncon, E. pers. comm. 2019), while *tregua* simply means “dog.” Some contemporary dogs in areas of central Chile, where indigenous populations persisted for several centuries after colonization, are “small ugly dogs,” with bulbous foreheads and eyes, and short floppy ears, but no curly hair (pers. obs. MR‐B). Perhaps these resemble the munutru or another indigenous dog morphotype. However, no specimens of Mapuche dogs are known to us.

The case of Patagonia in the extreme south of Chile also does not have good biological evidence about dogs. Ethnohistorical records of the 19th and 20th Centuries (Coiazzi, 1914) mention the presence of dogs in societies that had little or no contact with the Spanish or other colonists. Coiazzi documents the coexistence of *C. familiaris* with hunter‐gatherer societies of southern Patagonia, in particular the Selk'nam (also known as Ona), who used dogs to hunt guanacos among other animals. Martin Gusinde, famous Selk'nam ethnographer, also recognized the presence of, at least in some form, domesticated dogs in Selk'nam encampments. These dogs, described as loud, aggressive, and with a pointed snout (Gusinde, 1951), were very appreciated by the natives for their loyalty and protection, and as Gusinde described “In each house there are at least four of these acrimonious and irreconcilable dogs.” Gusinde also described Selk'nam using dogs to hunt guanacos, even apparently picking up and following a scent in packs, just like hunting scent hounds in the UK and other areas with a tradition in hunting (Gusinde, 1951). The initial Spanish colonization of Chile only extended to Chiloé which does not rule out a European origin of Selk'nam dogs via long‐distance trade, but also makes it plausible that these dogs had a previous origin in pre‐Columbian trade networks or migrations. Coiazzi (1914) suggests that the Selk'nam dogs had displaced the populations of a native canid similar to a fox, though whether he means by competition (cf. Vanak & Gompper, 2009) or as a favored domesticate is unclear. Carlos R. Gallardo (1910) also wrote about the relationship between Selk'nam people and domesticated, supposedly native, dogs, described as “*Canis (Pseudalopex) lycoides*,” also described as an important part of guanaco hunting. These domesticated dogs are described thoroughly in his book “The Onas”:

The fuegian dog is a not very big, wild looking animal. Some of them retain such a striking similarity to their ancestors, one can easily mistake them for a big fox, but (…) There are some of yellowish grey colour, of clear and almost white background, and with dark tints from black to ashen yellow. They have a broad forehead, straight, pointy, and fairly large ears, the eyes are somewhat oblique, the snout is long and even pointy, the neck is short, and the legs are notable for having very developed membranes between the fingers. The tail is long, covered in also long hair that coats the rest of the body as well.

**FIGURE 1:**
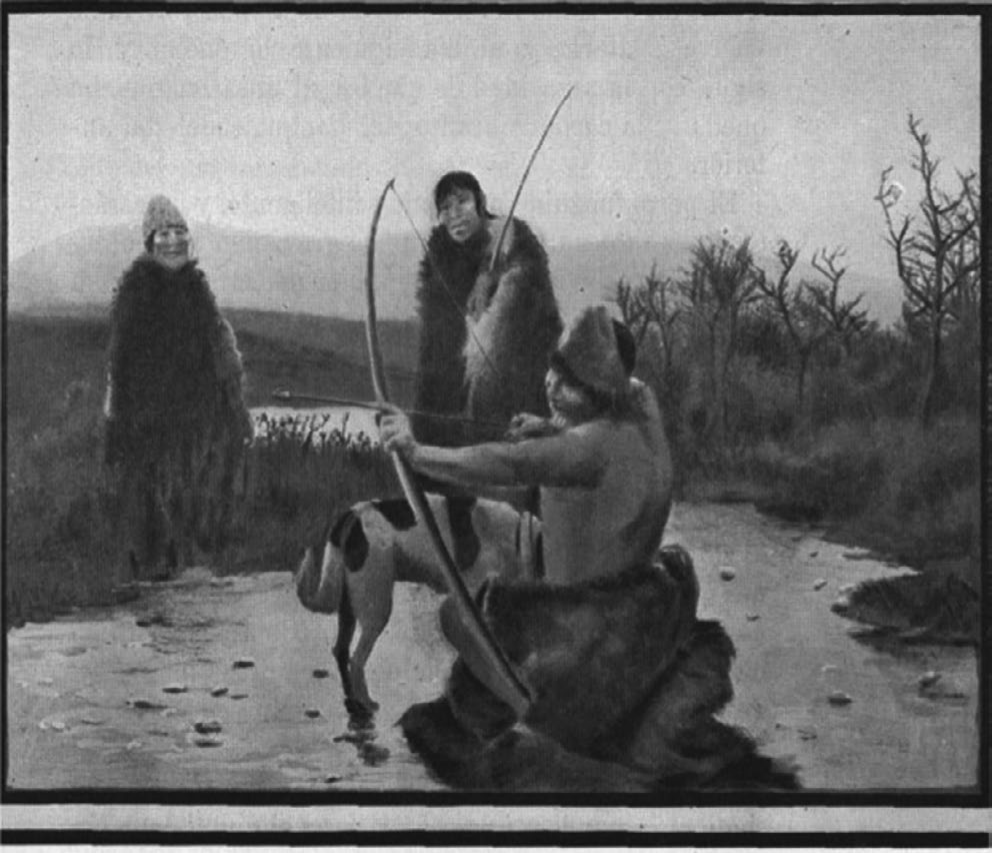
Reproduction of a drawing found in “The Onas” by Carlos Gallardo (1910) titled “Educating the dog” (*Educando al perro*). Some characteristics mentioned in the text can be seen in the drawing of this Fuegian dog. The image is free of copyright (http://www.memoriachilena.gob.cl/602/w3‐article‐8403.html)

Some of this description can be seen in Figure 1, adapted from drawings present in the same book (Gallardo, 1910). In this context and with such characteristics, the possibility of a native canid being domesticated in Tierra del Fuego by Selk'nam is certainly more plausible. In addition to this, the study by Petrigh and Fugassa (2013), in which they genetically identified a taxidermized canid belonging to native people of the area, showed that this specimen was closely related or identical to *L. culpaeus*.

### 2.5 Other, native, domesticated canids

Mitchell (2017) proposes a theory to explain the slow dynamics of spread of *C*. *familiaris* in South America, including the Southern Cone and Chile, after its original arrival across the Bering Strait. He argues that the canids of South America were better adapted to local diseases such as *Leishmaniasis canina* and distemper than *C. familiaris* and acted as reservoirs and vectors that led to almost epidemic levels of these diseases in dog populations, limiting their adoption by native peoples. The Andes may also have acted as a barrier to dog migration into Chile. An alternative perspective is developed by Prates (2014), who argues that many societies may have failed to incorporate *C*. *familiaris* into their social practices and daily lives if other canids already played roles such as keeping watch, hunting, or companionship. Prates bases this hypothesis on the discovery of a *Dusicyon avus* skeleton at a funerary site of the “Loma de los Muertos” archeological deposit in Río Negro Province, on the southern half of Argentina, dated around 2,000–3,000 ya (Prates, Prevosti, et al. , 2010; Stahl, 2012, 2013). This is most probably a deliberate burial. In the context of cosmological systems in many areas of South America in which taming of individual animals is a common practice and a fundamental part of their understanding of social relations between species (Erikson, 2000; Stahl, 1984), this suggests the hypothesis of possible taming of canids in many societies of the continent. It is thus not impossible to imagine that some sort of domestication or taming of native canids by Patagonian hunter‐gatherers happened in Chile (Gallardo, 1910; Gusinde, 1951). To be clear, taming is different from domestication in that taming is a behavior‐change process in which individual animals are socialized to live with humans, while domestication is an evolutionary process in which a population of an animal is selected for its ability to live with humans (among other features) (Gompper, 2014; Trut et al., 2009). Even domestic dogs, though generally showing a greater propensity for tameness, need to be properly socialized (Grandin & Johnson, 2006).

*Canis familiaris* was thus not necessarily the only canid either tamed or domesticated by humans. However, further research would be necessary to determine what kinds of relationships may have existed between native canids and humans in Chile, and to what extent this domestication took place, as at present we do not have archeological nor paleontological data to support these hypotheses.

### 2.6 Hunting strategies of and with canids in the Southern Cone and their effects on prey adaptations

It is difficult, based on morphology alone, to deduce behavioral strategies, given the amount of inter‐ and intraspecific variation in canids (Macdonald & Sillero‐Zubiri, 2004). Paleontologists, however, have noted that morphological specializations can favor particular hunting strategies in contemporary canids and felids, and thus, by analogy there may have been similar trade‐offs in extinct mammals (Andersson, 2005). For example, limbs can be cursorially specialized or specialized to “supinate the forearm, and thus grapple prey” when pouncing on it (Andersson, 2005 p. 57). When the prey cannot be effectively grappled with the forearms, bringing down large prey with the mouth is the alternative; this latter kill strategy favors pack hunting. Nevertheless, Andersson (2005) highlights exceptions. For example, lions, which can grapple excellently, are group hunters (using an ambush strategy), while *S*. *venaticus*, the bush dog, has very poor cursorial adaptations but is a group‐hunting species that brings prey down by biting. Ecological conditions such as shrub cover, by creating physical constraints, can also affect hunting strategies (Fanshawe & Fitzgibbon, 1993; Karanth & Sunquist, 2000; Thibault & Ouellet, 2005). Group hunting may contribute flexibility to hunting. Fanshawe and Fitzgibbon (1993), for example, show that African wild dogs, *Lycaon pictus*, unlike solitary ambush predators, were equally successful in killing prey in different amounts of cover, and alone or in groups of different sizes. While pack‐hunting cursorial species such as *C. lupus* typically “troll” for and “test” prey in a cursorial mode, they also have group‐hunting tactics that include ambushes (Fox, 1987). Many contemporary canid species hunt small prey alone and large prey in packs (e.g., *Canis lupus*, *C. latrans*, *C. adustus*, *S. venaticus*; Andersson, 2005).

The variation in predator behavior makes a difference to prey. When faced with ambush versus cursorial predators, large prey animals alter their antipredator behaviors, notably by being more vigilant in habitat types associated with stalking and ambushing predators (Makin et al., 2017). Preisser et al. (2007) conclude that predator identity, for example, hunting strategy, matters to prey life‐history trade‐offs.

*Dusicyon avus* is the only large Pleistocene canid for which we currently have positive evidence of its distribution in Chile (Castillo, 2007). Prevosti et al. (2009) report that *D*. *avus* likely predated *Lama* spp. as well as other large and medium animals, although they also indicate that *Lama* spp. may often have been scavenged rather than killed (Prevosti & Martin, 2013; Prevosti & Vizcaíno, 2006). The extinct *Dusicyon* spp. are described as “large foxes” (e.g., Prevosti et al., 2009), a description that may suggest that they did not live or hunt in packs, although like large‐fox‐like coyotes (*Canis latrans*) they might have lived in packs but hunted alone or in pairs. Coyotes are cursorial predators adapted to hunting in the open plains and prairies of North America (Thibault & Ouellet, 2005). At least part of the distribution of *Dusicyon* was open plains, so it could have shared a similar hunting strategy. Yet, as for *L*. *pictus* (Fanshawe & Fitzgibbon, 1993), they might be equally successful with a lone‐hunting cursorial strategy in areas with tree cover, at least for certain prey. The maned wolf, *Chrysocyon brachyurus*, is also large (up to 23 kg) (Sheldon, 1992) and described as fox‐like (Dietz, 1985), and in some accounts a sister genus of *Dusicyon* spp (Austin et al., 2013). While the maned wolf has never occurred in Chile to date (Torres et al., 2013), it would have coexisted with *Lama* spp., rheas (*Rhea* spp.), and other prey species whose ranges included Chile during the Holocene. It can thus both suggest potential *D. avus* hunting strategies by analogy, and suggest the kinds of strategies species found in Chile would also have been adapted to. Maned wolves hunt alone and usually at night (de Melo et al., 2007; Sheldon, 1992). They are typically found in open woodlands (Cerrado) and tropical grasslands. Maned wolves are opportunistic and flexible omnivores that eat a variety of plant parts, insects, small animals, and medium‐sized animals of a similar mass to its own, including armadillos, Pampas deer (*Ozotoceros bezoarticus*, 22–34 kg), and Greater rheas (*Rhea americana*, 20–27 kg) (Aragona & Setz, 2001; de Almeida Jácomo et al., 2004). These may be cases of scavenging, as there is only a single recorded observation of the maned wolf hunting and killing medium‐large prey (Bestelmeyer & Westbrook, 1998). The maned wolf, and *D. avus*, are considerably smaller than an adult guanaco (*L. guanicoe*), and the adults are thus very unlikely to have been actively hunted prey for these species. As for the culpeo fox, or coyotes in the context of llamas, we might expect that adult guanacos may have been able to counter‐attack lone‐hunting maned wolves as well as *D*. *avus*.

The surviving Pleistocene *Lycalopex* spp. found in Chile, as reported above, anecdotally attack juvenile camelids and prey on entrapped adults. However, presumably it is difficult for the lone‐hunting *Lycalopex* spp. to bring down adult guanacos or native deer, due to their small size, and their lack of a group‐hunting strategy (cf. the equally small bush dogs, *Speothos venaticus*, which are only able to bring down deer by biting them in large packs) (Biben, 1982; Sheldon, 1992). Otherwise, *Lycalopex* spp. can prey on fawns of medium and large ungulates (Corti et al., 2010).

We cannot necessarily conclude that widely distributed species such as guanacos, which would have been exposed prehistorically to cursorial pack‐hunting, had different antipredator adaptations in the populations to the west and the east of the Andes. However, it is possible that with the only remaining pack‐hunting species in South America being the bush dog in the Amazonian basin, prey in the Southern Cone like guanaco may have lost, since the Pleistocene extinctions of the hypercarnivorous species, adaptations specific to surviving group predation tactics, whether cursorial or ambush.

By contrast, domesticated dogs may have been incorporated into particular roles in hunting with humans. Lupo (2017) presents ethnographic evidence that of the many forms of hunting with dogs, most of them, under most circumstances, have no appreciable benefit for hunting success. She argues that dogs are least useful for large prey that need to be hunted by stealth, and often interfere in ambushes and traps, but can be useful for finding and flushing prey, and handling certain prey. Although a large increase in effectiveness of hunting (compared to hunting without dogs) is observed when dogs are introduced as novel predators to islands and used in packs in combination with guns, other factors such as colonialism and land‐use change also simultaneously alter technologies, economic drivers, and environmental conditions, such that the role of dogs alone is unclear in driving hunting outcomes (Lupo, 2017). We cannot assume that even if hunting with larger dog packs might be more effective, hunters would necessarily prefer this tactic, since hunter‐gatherers and many agriculturalists do not purposefully maximize productivity (Sahlins, 1974[2017]). Rather, the evidence from Lupo (2017) is more consistent with viewing dogs as part of the social group (see the discussion of taming, above), than as hunting tools. How, in fact, were dogs incorporated into hunting tactics in Chile and the Southern Cone?

The dogs of the northern Andean civilizations and the north of Chile are all rather small, but some of them may have been used to corner game. The *munutru* was reportedly also small. The dogs of the Selk'nam, which appear to be medium‐sized and shaped like dingoes (Alvarado et al., 2007), had roles including chasing down guanacos that ran off after being stalked and struck by an arrow, taking down guanacos that the hunters were ambushing, or helping chase guanacos toward an ambush site (Legoupil, 2011). Belardi et al. (2017) describe early Holocene communal hunting strategies to the East of the Andes, in Patagonia, that involved one group of hunters driving guanacos toward another group lying in wait; later on, constructed blinds were used. Santiago and Salemme (2016) report that in addition to using group ambush tactics such as those described above, Selk'nam men often hunted alone, and women sometimes hunted with dogs: They do not clarify whether this was by pursuit or stalking. Across the Americas and across the Holocene, communal hunting driving large prey toward ambushes, opportunistic hunting of large animals mired in natural “traps” such as bogs and tar pits, and building traps, including nets, are among the many tactics thought to have been used, with or without dogs (Davis & Reeves, 2014). The use of trapping technologies and tactics can be seen as a kind of displaced ambush, which like other forms of ambush require the prey to be highly alert and discerning about danger cues that try to blend into the environment (Gell, 1996). We have not found clear evidence, before horses and cars, of pursuit followed by killing as a human hunting tactic for guanaco or other prey in Chile and the Southern Cone. Consequently, if indeed human hunting with dogs to the west of the Andes and the extreme south of the continent predated Europeans (as seems plausible), in itself this does not mean that guanacos or other prey were exposed to the same selective pressures as pack‐hunting cursorial feral and free‐ranging dogs pose today.

## 3 CONCLUSIONS

Why are the key large and medium prey species of Chile—camelids and deer—apparently unadapted to cursorial pack hunting by contemporary feral and free‐ranging dogs? In summary, west of the Andes (within Chile) there is no clear evidence of group‐hunting species or cursorial specialists large enough to bring down adults of these species (with the possible exception of the very small pudu *Pudu puda*). However, the guanaco in particular has had a range throughout the Southern Cone and should have been exposed to Pleistocene pack‐hunting species that would have used cursorial strategies. It is unclear evolutionarily speaking whether appropriate antipredation strategies might have been found only in the guanaco populations exposed to these predation pressures east of the Andes. It remains unclear, for lack of data, whether extant populations of guanacos east of the Andes are differently adapted to dog predation; dogs are not described as a primary threat or major predator in literature on guanacos in Argentina. The biology and evolution of predation defense suggests that suites of different kinds of defense behaviors can be maintained as long as any kind of predators are present (Blumstein, 2006). Since there have always been puma and foxes in Chile, we would thus expect the prey species to maintain the capacity for any antipredator behaviors that they had to evolve.

For guanacos with their wide distribution in particular, we can thus outline three possible scenarios or hypotheses: (1) Only certain guanaco lineages east of the Andes were adapted to group‐hunting cursorial strategies, and these populations have gone extinct (or are isolated from the Chilean populations); (2) the entire species had these adaptations but have lost them with the extinction of the predators in question at the end of the Pleistocene, contrary to the implication of Blumstein (2006); (3) these adaptations are latent in all guanacos' repertoire of possible adaptative responses to predation but for some reason which remains unclear seem to be expressed inadequately in Chilean populations despite existing predation pressure. As for deer species and other camelids of Chile, these species have smaller ranges largely restricted to the Andes and the west of the Andes, so their prehistoric exposure to group‐hunting cursorial strategies of predators east of the Andes is less clear, but possibly null.

However, these hypotheses are complicated by also considering the factor of indigenous people's pre‐Columbian hunting strategies involving domesticated canids, whether dogs or other species. It is probable that human hunters throughout the Southern Cone including Chile used dogs to assist in hunting well before the arrival of Europeans. These strategies were probably, given historical and comparative ethnographic evidence, primarily ambush strategies, although we cannot totally rule out cursorial‐type strategies since group hunting in canids (and in humans) is usually quite flexible in form. So, we suggest two further hypotheses, which both take into account human hunting with dogs, and the question about latency of adaptations raised in hypothesis (3). (4) Pre‐Columbian indigenous hunters also had an advantage in killing prey with canids similar to what is currently observed in canid hunting in Chile today, and for some reason, prey species largely did not adapt to this group‐hunting attack strategy. This lack of adaptation could be directly related to the variability in both human and dog hunting strategies, as continues to be observed in feral and free‐ranging dogs today. It might also be due to lack of historical depth or continuity of the practices, or to a continually changing habitat structure under human influences (which would affect predation strategies and success rates); (5) prey species did adapt to the group‐hunting human–dog strategy, but only to the dominant ambush strategies, which is why they remain vulnerable to cursorial attacks. Again, it is not totally clear from the biology of adaptation to predation whether these two antipredation strategies can be neatly separated in this way (adaptation to one but not the other), since most examples of flexible prey antipredation adaptations come from prey exposed to both ambush and cursorial strategies; we are not aware of examples of nonflexible prey antipredation adaptations in populations exposed to both strategies. This is an implicit subhypothesis that requires further research.

Although the logic of Geist (1998) suggesting a lack of cursorial predators is persuasive to explain deer adaptations, when also considering camelids and when carefully looking at the evidence, the situation appears to be more complex than his argument accounts for.

We conclude that our state of knowledge about the biology and evolution of prey adaptations to different predation strategies, and the state of the evidence base for the existence of different canids in Chile and the rest of the Southern Cone of South America, are inadequate to address how the problem of feral and free‐ranging dog predation on native species in these regions might be solved. For example, if native prey species really have no evolutionary background of exposure to group‐hunting cursorial species, then only a natural process of selection could lead to its emergence. Whether it would be better, or more feasible, to expose native prey species to feral and free‐ranging dogs in the hope of their eventual adaptation, or to eradicate feral and free‐ranging dogs, is an open question. On the other hand, if guanacos in particular, and perhaps other species, have latent adaptations to group‐hunting cursorial species, it is somewhat mysterious as to why they are so often killed by feral and free‐ranging dogs. Here, there may be two more hypotheses to consider, which are quite different. (6) It could be the case that the rate of feral and free‐ranging dog hunting success is not exceptionally high compared with “natural” predation, but is problematized due to its seeming unnaturalness. With current information, this is difficult to assess. (7) It could be that camelids and deer need to learn socially (from parents or others) how to use certain antipredator strategies of which they are behaviorally capable (Wiedenmayer, 2009), but this possibility has been lost at some point in history due to loss of the behavioral expression among Chilean populations, either due to a Pleistocene predator extinction, or due to the local discontinuation of human hunting with dogs among relictual deer and camelid populations. In the latter case, training individuals to respond correctly to cursorial group attacks, or in situ exposure to predator densities below the coexistence threshold (Blumstein et al., 2019; Griffin et al., 2000), could be an approach to mitigate the problem by allowing social learning to spread.

Although we do not offer any answers here, we have clearly outlined the existing relevant knowledge and developed an array of hypotheses that should be tested, both to advance general knowledge of adaptations to predation and to assist conservationists in designing appropriate interventions to conserve camelids and deer in South America and Chile in particular.

## CONFLICT OF INTEREST

The authors declare no conflict of interest.

## AUTHOR CONTRIBUTION

**Benjamín Silva Rochefort:** Conceptualization (supporting); Investigation (lead); Writing‐original draft (lead); Writing‐review & editing (supporting). **Meredith Root‐Bernstein:** Conceptualization (lead); Investigation (supporting); Supervision (lead); Writing‐review & editing (supporting).

## Data Availability

This is a qualitative review and analysis, and there are no original or re‐analyzed data to share beyond the text found in this document.
